# Melanoma dormancy in a mouse model is linked to GILZ/FOXO3A-dependent quiescence of disseminated stem-like cells

**DOI:** 10.1038/srep30405

**Published:** 2016-07-28

**Authors:** Yasmine Touil, Pascaline Segard, Pauline Ostyn, Severine Begard, Caroline Aspord, Raja El Machhour, Bernadette Masselot, Jerome Vandomme, Pilar Flamenco, Thierry Idziorek, Martin Figeac, Pierre Formstecher, Bruno Quesnel, Renata Polakowska

**Affiliations:** 1 Inserm UMR-S1172 Centre de Recherche Jean Pierre Aubert (JPArc), Institut pour la Recherche sur le Cancer de Lille (IRCL), 1 Place de Verdun, 59045 Lille, France; 2 Faculté de Médecine, Université de Lille, 59000 Lille, France; 3 SIRIC ONCOLille, Lille, France; 4 Institut A. Bonniot, Ontogenèse et Oncogenèse Moléculaires, UMR EFS-UJF-INSERM 823, Immunobiology and Immunotherapy of Cancers, Grenoble, France; 5 Faculté de Chirurgie Dentaire, Université de Lille, 59000 Lille, France; 6 Plateforme de Génomique Fonctionnelle, IRCL, 59045 Lille, France; 7 Cancéropôle Nord-Ouest, 6 rue du Professeur Laguesse, 59045 Lille, France; 8 Service des Maladies du Sang, CHRU de Lille, 59037 Lille, France

## Abstract

Metastatic cancer relapses following the reactivation of dormant, disseminated tumour cells; however, the cells and factors involved in this reactivation are just beginning to be identified. Using an immunotherapy-based syngeneic model of melanoma dormancy and GFP-labelled dormant cell-derived cell lines, we determined that vaccination against melanoma prevented tumour growth but did not prevent tumour cell dissemination or eliminate all tumour cells. The persistent disseminated melanoma tumour cells were quiescent and asymptomatic for one year. The quiescence/activation of these cells *in vitro* and the dormancy of melanoma *in vivo* appeared to be regulated by glucocorticoid-induced leucine zipper (GILZ)-mediated immunosuppression. GILZ expression was low in dormant cell-derived cultures, and re-expression of GILZ inactivated FOXO3A and its downstream target, p21CIP1. The ability of dormancy-competent cells to re-enter the cell cycle increased after a second round of cellular dormancy *in vivo* in association with shortened tumour dormancy period and faster and more aggressive melanoma relapse. Our data indicate that future cancer treatments should be adjusted according to the stage of disease progression.

After presumably successful therapy, cancer patients, and even healthy people^1^, contain tumour cells nested in their organs and/or circulating in systemic fluids that remain asymptomatic for an extended period of time. These dormant cells have no apparent immediate potential to develop into clinically manifested tumours until they are activated by mechanisms that have not yet been well defined. Even if cells exit dormancy and a dividing tumour cell population expands, the tumour mass may not reach a detectable size, in which case disease relapse would not occur. Clinically, this phenomenon is referred to as tumour dormancy or minimal residual disease. Although the outcomes of cellular dormancy and tumour dormancy are related, they are mechanistically different events. Cellular dormancy appears to be regulated at the single-cell level during division, and it may be part of the mechanism responsible for tumour dormancy. The tumour dormancy process is most likely controlled by the immune system and “angiogenic switch” mechanisms, both of which balance the processes of cell proliferation, differentiation and death to determine the net size of a tumour cell population^2^,^3^,^4^,^5^,^6^,^7^. Recent accumulated evidence suggests that dormant cells may display stem cell (SC) properties^5^,^8^,^9^,^10^. Moreover, cellular quiescence appears to be one of the major mechanisms preventing SC exhaustion and protection from adverse environmental conditions^8^,^11^. However, whether tumour SC quiescence is responsible for tumour dormancy and whether the reactivation of quiescent cells is linked to tumour relapse is not yet certain. It is also unknown whether the factors that reactivate dormant cells are the same factors that cause tumour relapse. In the present study, we developed a murine tumour dormancy model of melanoma to investigate the roles of cellular quiescence and related factors in a potential mechanism of tumour dormancy.

Previously, we have shown that inhibition of the PI3K/AKT signalling pathway reactivates a quiescent, and inactivates a cycling, subset of melanoma SCs (MeSCs), demonstrating that this pathway differentially regulates both quiescent and cycling MeSCs^12^. The PI3K/AKT pathway, which is often deregulated in cancers^3^,^13^ including melanomas^12^,^14^,^15^, is involved in the maintenance of normal and cancer SCs (CSCs) and tissue/tumour regeneration and is therefore essential for SC self-renewal and survival^16^. In dormant DA1-3b acute myeloid leukaemia cells^17^,^18^, AKT is down-regulated by glucocorticoid-induced leucine zipper (GILZ), an essential mediator of glucocorticoid activity^19^,^20^,^21^ encoded by *Tsc22d3* gene that is responsible for the regulation of FOXO3A, resistance to anticancer drugs^18^, and pro-apoptotic functions^18^,^20^,^21^. Here, we provide evidence that persistent disseminated melanoma cells (DMCs) display SC properties and that GILZ controls their quiescent and activated states, which are closely linked to clinically observed melanoma dormancy and relapse.

## Results

### Isolation of quiescent and immune-resistant DMCs from a mouse model of melanoma tumour dormancy

**Figure 1:**
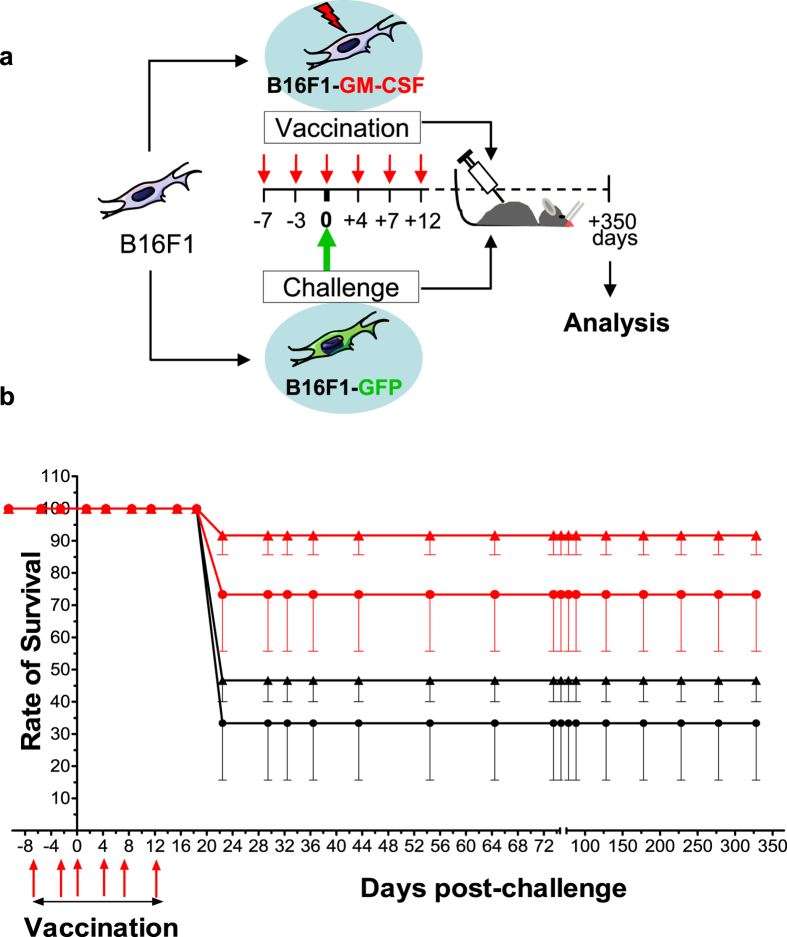
Vaccination with irradiated B16F1 murine melanoma cells expressing GM-CSF elicited anti-melanoma activity and improved survival of C57BL6/rj mice. (**a**) Schema of the vaccination protocol: C57BL6/rj mice were injected i.d. twice a week for 19 days (red arrows) with 1 × 10^6^ irradiated B16F1 cells either expressing GM-CSF (B16F1-GM-CSF) or carrying an empty pcDNA3.1-Zeo vector, (not shown for clarity). The mice were challenged (green arrow) with B16F1 cells expressing GFP (B16F1-GFP, s.c. injection) at one week after initiation of the vaccination protocol, and the organs of surviving mice were analysed 350 days later. (**b**) Survival rates of 40 mice injected with 1 × 10^6^ irradiated B16F1-pcDNA3.1-Zeo control cells (black) and 55 mice injected with 1 × 10^6^ irradiated B16F1-GM-CSF cells (red) after challenge with 2 × 10^3^ (▲) or 5 × 10^3^ (●) B16F1-GFP cells. The results are presented as the means ± SEM of 3 independent experiments. The red arrows indicate the days when the mice were vaccinated (7 and 3 days before the challenge, indicated as “0”, and 4, 7 and 12 days after the challenge).

To obtain insights into the mechanisms underlying tumour dormancy, we established a syngeneic mouse model of dormancy using melanoma-based immunotherapy (Fig. 1a) by modifying a previously described protocol for generation of a DA1-3b acute myeloid leukaemia model^17^. The mice were vaccinated with irradiated B16F1 murine melanoma cells expressing recombinant mouse granulocyte-macrophage colony-stimulating factor (B16F1-GM-CSF), which is known to confer anti-melanoma protection in animal models^22^,^23^. The mice were pre-immunised 7 days before immune challenge with native B16F1-GFP cells and were then immunised twice a week for 12 days during immune challenge (Fig. 1a). These mice exhibited significantly improved survival for a minimum of 350 days compared with control mice, which were engrafted with irradiated B16F1-pcDNA3.1-Zeo cells (Fig. 1b and Supplementary Table S1). These results indicated that GM-CSF was bioactive and inhibited tumour development, as expected based on previous reports^23^.

Because the accumulated data suggest that tumour cells disseminate early, even prior to development of the primary tumour^24^,^25^, we examined the organs of tumour-free mice for the presence of dormant B16F1-GFP melanoma cells. qRT-PCR revealed that 35.5% (6/17) of the immunised mice and 33.3% (3/9) of the control mice contained GFP-positive (GFP+) cells that had disseminated to various organs without any consistent pattern (Supplementary Table S2). These cells mainly disseminated to the brain, liver and skin, which are the well-known metastatic sites of relapsed melanoma^26^. The lack of visible tumour masses in these organs indicated that the DMCs were unable to colonise and develop into tumours within 350 days in either the control or immunised mice. Taken together, these data showed that vaccination against melanoma prevented tumour growth but did not prevent tumour cell dissemination or eliminate all tumour cells. Surviving DMCs remained asymptomatic and thus dormant in multiple organs. These cells clearly evade even the GM-CSF-stimulated immune system, which generally reinforces protection against B16 melanoma by increasing antigen presentation and T-cell priming capabilities^23^. CSCs have been shown to be less antigenic than their differentiating progeny, which comprise the majority of tumour masses^5^,^27^. This evidence suggests that the DMCs that evaded elimination in this study may be MeSCs.

### Dormant melanoma cells display CSC properties

**Figure 2:**
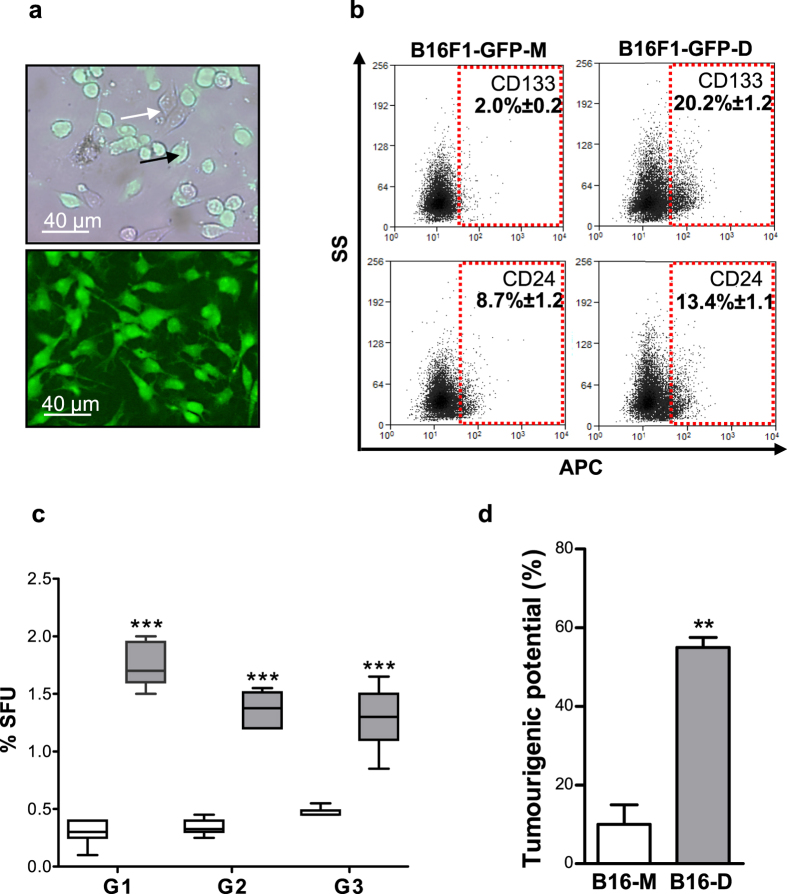
The dormant B16F1-GFP cell line was enriched in melanoma cells with stem cell properties. (**a**) Fluorescent microscopic images of FACS-sorted GFP-positive dormant cells isolated from organs of tumour-free mice, cultured for 23 (up) days and then cloned (down). The white and black arrows indicate GFP-negative and GFP-positive cells, respectively. (**b**) Flow cytometry dot plot profiles of the stem cell surface markers, CD133 (up) and CD24 (down), expressed by both the maternal B16F1-GFP-M (left) and dormant DMC-derived B16F1-GFP-D (right) clonal cell lines. The numbers represent the mean percentage ± SEM. (**c**) Box plot of sphere-forming units (SFUs) formed by B16F1-GFP-M (white) and B16F1-GFP-D (grey) cells plated at a clonal density of 1000 cells/ml. SFUs were calculated according to the following formula: % SFU = number of spheres/number of plated cellsx100. Self-renewal ability was evaluated by dissociating the spheres and then re-plating the sphere cell suspensions for 3 successive sphere generations (G1–G3). The graphs represent 3 independent experiments performed in quadruplicate. ***p < 0.001. (**d**) Dormant DMC-derived B16F1-GFP-D cells (B16-D) had a higher tumourigenic potential than maternal B16F1-GFP-M cells (B16-M). The mice were injected s.c. with 200 B16F1-GFP-M or B16F1-GFP-D cells. The results are expressed as the per cent of mice bearing tumours to the total number of injected mice. The histograms represent the means ± SEM of 2 independent experiments. **p < 0.01.

To determine the identities and properties of the surviving B16F1-GFP DMCs, 100 to 40,000 GFP+ cells (depending on the experiment) were isolated via fluorescence-activated cell sorting (FACS) from the organs of tumour-free mice at one year after the challenge. Among several independent trials, only one sample of GFP+ cells isolated from pooled organs of the control and immunised mice, which are listed in Supplementary Table S2, divided to form small colonies after 3 weeks of latency (Fig. 2a). We cloned the GFP+ cells after expansion. Only one of these colonies generated a dormant DMC-derived B16F1-GFP-D cell line. The term “dormant” refers to a founding DMC that persisted *in vivo* for a long period of time without clinical manifestations. The poor efficiency in obtaining GFP+ dormant DMC-derived cell lines indicates that the long-term dormant cells *in vivo* were proliferation resistant *in vitro* for several weeks. The ability of *in vivo* dormant GFP+ cells to ultimately proliferate and to reconstitute the cellular heterogeneity of the dormant DMC-derived cell line, suggests that *in vivo* asymptomatic, dormant DMCs have characteristics of reversibly quiescent CSCs. This finding was supported by the results of flow cytometry analysis (Fig. 2b), which showed that the reactivated dormant DMC-derived B16F1-GFP-D population was enriched in cells overexpressing the surface markers CD133 and CD24, which are associated with the B16 MeSC phenotype^28^, compared to the non-dormant “maternal” B16F1-GFP-M cells. Functionally, the B16F1-GFP-D cells formed 5.7 times more self-renewing spheres than the B16F1-GFP-M cells (Fig. 2c), reflecting the larger size of the SC pool in the B16F1-GFP-D cultures. The size of the CSC cell population is correlated with tumour aggressiveness^29^. Further, the *in vivo* tumourigenic potential of the B16F1-GFP-D cells was found to be consistently higher than that of the B16F1-GFP-M cells (Fig. 2d) because as few as 200 B16F1-GFP-D cells initiated tumour development in 57.1% (8/14) of the immunocompetent C57BL6JRj mice. By contrast, the same number of B16F1-GFP-M cells initiated tumour growth in only 14.3% (2/14) of the mice. This finding demonstrated that the dormant DMC-derived cell line contained a higher proportion of tumour-initiating MeSCs than the maternal cell line. Based on these results, we conclude that among DMCs, only replication-quiescent MeSCs are capable of long-term survival *in vivo* and that the GM-CSF-stimulated immune system, which appears to effectively prevent the formation of primary tumours, is ineffective in eradicating these dormant/quiescent MeSCs. Additional evidence supporting this conclusion was provided by the proof-of-concept experimental data obtained using a human melanoma dormancy model (Supplementary Fig. S1), which demonstrated that the immune system of our “humanised” mouse model^30^ inhibited the growth of a primary human melanoma tumour but was unable to eliminate human MeSCs, which were identified according to their H2B-GFP label-retaining properties^31^,^32^ and which disseminated to multiple organs (Supplementary Table S3). Therefore, it appears that asymptomatic DMCs that persist at multiple metastatic sites are quiescent MeSCs preselected by evasion of immune responses. These quiescent MeSCs established tumour dormancy *in vivo* in our clinically relevant syngeneic murine melanoma model. In addition, the results obtained by xenografting quiescent human MeSCs into our “humanised” mouse model suggest that a similar mechanism may operate in humans.

### Re-entry into metastatic dormancy preselects persistent DMCs with new properties that facilitate their propagation *in vitro*

**Figure 3:**
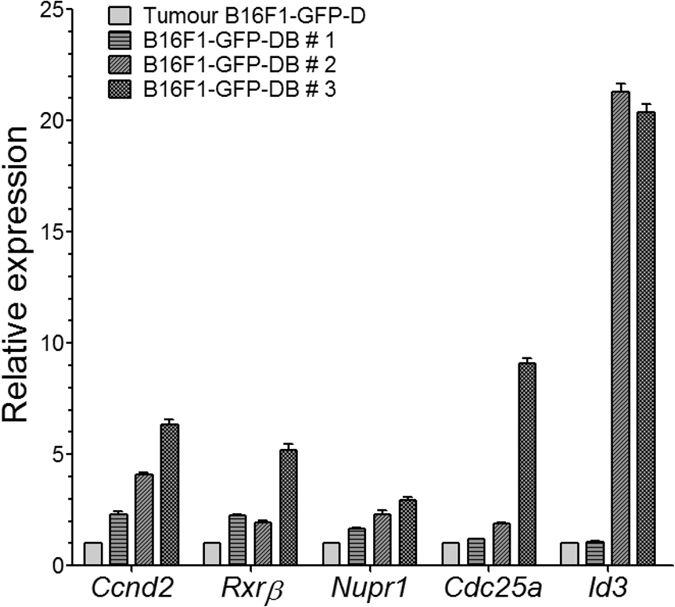
The brain microenvironment preselected dormant DMC-derived cells with a new phenotype. Dormant DMC-derived B16F1-GFP-D cells persisting asymptomatically in the brains of mice bearing primary tumours overexpressed factors that potentially facilitated their *in vitro* propagation. RT-PCR was performed to analyse *Ccnd2, Rxrb, Nupr1, Cdc25a* and *Id3* expression in B16F1-GFP-DB #1, #2 and #3 brain-derived GFP+ cells and in their respective primary tumours #1, #2 and #3. The values represent the levels of each transcript in the brain-derived B16F1-GFP-DB cells relative to the average levels of the corresponding transcripts (set to 1) in three B16F1-GFP-D-initiated tumours.

CSCs are responsible for reconstitution of the original tumour/culture cellular heterogeneity and tumour relapse^8^,^33^. We detected GFP+ cells by qRT-PCR (Supplementary Table S4) and cultured them cells from different metastasis-free organs obtained from mice bearing primary melanoma tumours established by either B16F1-GFP-D or B16F1-GFP-M cells. After 4 weeks of latency, as many as 66.6% of the B16F1-GFP-D cells disseminated to the brain were capable of forming colonies *in vitro*, thereby creating a second generation of brain-derived dormant (B16F1-GFP-DB) melanoma cell lines. Cells isolated from other organs and from the brains of mice injected with maternal B16F1-GFP-M cells never formed colonies. This observation demonstrated that, in the brain microenvironment, the *in vivo* asymptomatic B16F1-GFP-D MeSCs acquired the capacity to readily form colonies *in vitro* and indicated the intrinsic differences between dormant DMC-derived and maternal cell lines. Pairwise comparative RT-PCR analysis (Fig. 3) of B16F1-GFP-D cell-derived tumours and the respective brain-derived B16F1-GFP-DB cell lines revealed that the B16F1-GFP-DB cells exhibited consistent overexpression of some of the 248 genes that were differentially regulated between the dormant cell-derived and maternal cell lines (Supplementary Fig. S2a), such as cyclin D2 (*Ccnd2*), a cell cycle control gene responsible for asymmetric self-renewal of SCs^34^,^35^; cell division cycle 25A (*Cdc25a*) a phosphatase controlling cell cycle progression^36^; *Nupr1* (p8), which regulates p21CIP1 and tumour progression^37^; retinoic acid X receptor β (*Rxrb*), which modulates immune responses in melanoma^38^; and inhibitor of differentiation (*Id3*), which regulates p21CIP1 and drives CSC self-renewal^39^. The same results were obtained with the three independent tumour/brain cell pairs. These findings excluded the possibility that *de novo* mutations were present in each individual mouse and led to the conclusion that the brain microenvironment either pre-selected B16F1-GFP-DB founder cells from a pre-existing sub-clone of the B16F1-GFP-D culture or that it induced certain epigenetic alterations; further, these findings suggested that overexpression of the *Ccnd2, Rxrb, Nupr1, Cdc25a* and *Id3* genes facilitated *in vitro* propagation of the B16F1-GFP-DB cells. In conclusion, each generation of persistent DMCs appeared to acquire new properties, which increased their ability to re-enter the cell cycle. This finding is consistent with clinical observations that relapsed metastatic tumours are more aggressive than primary tumours.

### Cellular quiescence of dormant DMC-derived melanoma cells is correlated with low GILZ expression

**Figure 4:**
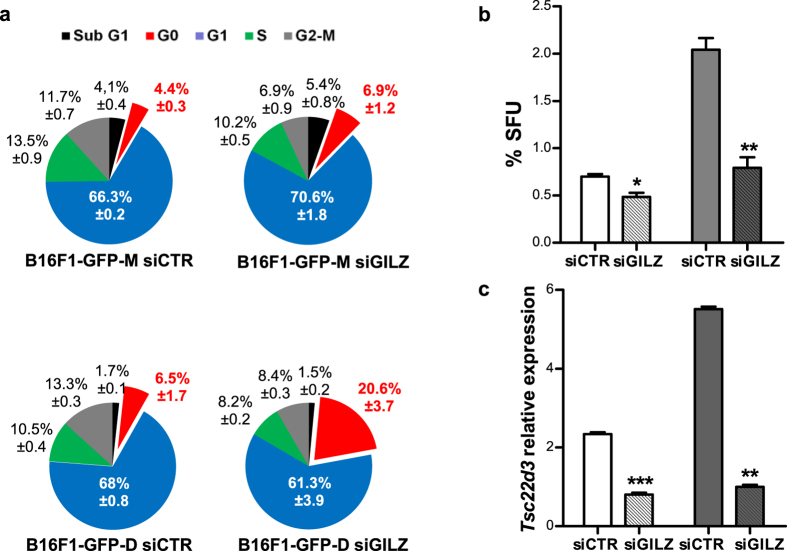
Down-regulation of GILZ expression induced quiescence of dormant DMC-derived B16F1-GFP-D cells in the G0 phase. (**a**) Cell cycle analysis of dormant DMC-derived B16F1-GFP-D (lower panel) and maternal B16F1-GFP-M (upper panel) cells transfected with control (left) or GILZ-specific (right) siRNA revealed that the dormant DMC-derived cells possessed the novel, GILZ-dependent ability to control the G0-to-G1 transition in addition to the known GILZ-dependent ability to control the G1-to-S transition of the cell cycle. A fraction of cells in the G0 phase of the cell cycle are shown in red. (**b**) Sphere-forming units (SFUs) formed by B16F1-GFP-D (grey) or B16F1-GFP-M (white) cells transfected with control and GILZ-specific siRNA. (**c**) qRT-PCR assay of *Tsc22d3* expression in spheres obtained from B16F1-GFP-M and B16F1-GFP-D cells transfected with either control or GILZ-specific siRNA and cultured for 7 days. The values indicate the level of GILZ-encoding mRNA relative to the control (B16-F1GFP-M adherent cells) (ΔΔCt); siCTR (control siRNA), siGILZ (GILZ-specific siRNA). The results represent 3 independent experiments. *p < 0.05; **p < 0.01; ***p < 0.001.

Cellular dormancy or mitotic quiescence is maintained by incompatible microenvironments and dormancy-associated genes^2^,^10^,^40^. To explore the molecular mechanisms responsible for melanoma dormancy, we performed comparative transcriptome analysis of dormant DMC-derived B16F1-GFP-D and maternal B16F1-GFP-M cells. A total of 248 genes were found to be differentially expressed between these two cell lines (Supplementary Fig. S2a, accession number GSE69703). Ingenuity Pathway Network Analysis (Fisher’s test ≤ 0.01) revealed that the down- and up-regulated genes in the dormant DMC-derived B16F1-GFP-D cell line were involved in cell cycle regulation, DNA repair, metabolism and carcinogenesis (Supplementary Fig. S2b). Among the genes down-regulated in the dormant cell-derived B16F1-GFP-D cultures was *Tsc22d3* (log −3.29), which has also been shown to be down-regulated in a dormant DA1-3b murine acute myeloid leukaemia cell line^17^. Interestingly, 3 genes, *Ccnd2, Cdc25a*, and *Id3*, which were overexpressed in the second-generation B16F1-GFP-DB cells, participate in a putative signalling network interconnected with the *Tsc22d3* regulatory circuit (Supplementary Fig. S3a). Because the *Tsc22d3* gene encodes the GILZ protein^20^, which mediates the anti-inflammatory and immunosuppressive effects of glucocorticoids^21^,^41^, and because immune responses control melanoma dormancy^24^,^25^, we anticipated that GILZ may be an important regulator of tumour dormancy. Both qRT-PCR and western blot analyses confirmed that the dormant DMC-derived cells expressed a considerably lower level of GILZ than the maternal cells (Supplementary Fig. S3b). These results suggest that GILZ may antagonise cellular quiescence in murine melanoma. This possibility was confirmed by cell cycle analysis, which showed that the lower *Tsc22d3* level in B16F1-GFP-D cells compared with B16F1-GFP-M cells was correlated with a higher proportion of cells arrested in the G0/G1 phase (Fig. 4a). Interestingly, knockdown (KD) of GILZ expression using GILZ-specific siRNA resulted in the massive transition of dormant DMC-derived B16F1-GFP-D cells from the G1 phase to the G0 phase of the cell cycle (Fig. 4a). By contrast, this transition was insignificant in the B16F1-GFP-M cells and occurred mainly at the expense of their S/G2/M cycling counterparts. Moreover, GILZ KD specifically decreased the number of replicating, ethynyldeoxyuridine (EdU)-incorporating CD133+ cells (Supplementary Fig. S5a) in the B16F1-GFP-D cultures but did not significantly change their total number in either the maternal or control dormant cell-derived cultures (Supplementary Fig. S5b). This finding suggests that whereas GILZ KD does not affect cell surface CD133 expression, it does inhibit the proliferation of dormant DMC-derived CD133+ cells. These data have further demonstrated the phenotypic differences between dormant DMC-derived cells and non-dormant cells and revealed that GILZ regulates the G0-to-G1 transition, most likely by promoting the exit of dormant melanoma cells from G0 reversible quiescence.

Reversible quiescence is a characteristic of SCs^5^,^8^,^11^. Interestingly, the increased number of B16F1-GFP-D cells that entered the G0 phase of the cell cycle in response to GILZ down-regulation was directly proportional to the increased size of the MeSC pool in the dormant cell-derived cultures, as judged by the superior sphere-forming capacity of the B16F1-GFP-D cells (Figs 2c and 4b) relative to the maternal cells. These results suggest that the massive amount of cells entering the G0 phase in response to GILZ KD were G1-arrested MeSCs that were consequently unable to form spheres (Fig. 4b). Consistently, dormant DMC-derived sphere cultures that were still mainly composed of live quiescent cells (not shown) on day 7 exhibited a low level of GILZ expression (Fig. 4c). These data demonstrate that GILZ is required for the *in vitro* propagation and sphere formation of MeSC that are dormant *in vivo*, and these findings suggest that GILZ re-expression may be responsible for tumour formation *in vivo* in a manner similar to its role in sphere formation *in vitro*. Conversely, GILZ inactivation may induce tumour dormancy *in vivo*, as it abolished tumour-like sphere formation *in vitro*.

### GILZ KD delays tumour formation and diminishes tumour growth but increases the tumourigenic potentials of mouse and human melanoma cells

**Figure 5:**
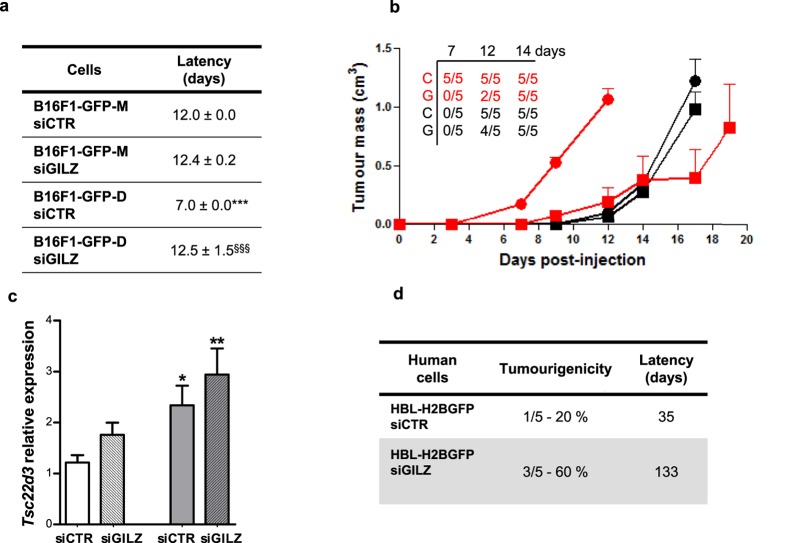
GILZ knockdown delayed tumour formation and increased tumourigenicity in syngeneic mice. Twenty C57BL6/rj mice were injected (s.c.) with 5 × 10^3^ B16F1-GFP-M or B16F1-GFP-D cells after transient transfection with control siRNA (siCTR) or GILZ-specific siRNA (siGILZ). (**a**) Delayed tumour appearance (mean ± SEM) was observed under the indicated conditions (n = 5). ***p < 0.001 vs. B16F1-GFP-M siCTR cells; ^§§§^p < 0.001 vs. B16F1-GFP-D siCTR cells. (**b**) Graph showing the tumour sizes at each time point after transient transfection with control (●) or GILZ-specific (■) siRNA in B16F1-GFP-M (black) and B16F1-GFP-D (red) cells. The numbers in the upper left corner of the graph indicate the number of mice with tumours/the total number of mice; C = control siRNA, and G = GILZ-specific siRNA. Tumour size was evaluated as described in the Methods. (**c**) qRT-PCR assay of *Tsc22d3* expression in B16F1-GFP-M (white) and B16F1-GFP-D cells (grey) transfected with control (siCTR) or GILZ-specific (siGILZ, stripes) siRNA. **p < 0.01; *p < 0.05 (n = 5). (**d**) The percentage of mice bearing human HBL melanoma tumours and the delay in tumour appearance. CB17-SCID mice were injected (s.c.) with 5 × 10^6^ HBL H2B-GFP cells transfected with either control or GILZ-specific siRNA (5 mice per condition).

To determine whether GILZ repression affects tumour formation *in vivo*, we knocked down GILZ expression in B16F1-GFP-D dormant DMC-derived cells and B16F1-GFP-M maternal cells using siRNA technology. Then, 24 h later, we injected 10,000 cells from each cell line into syngeneic C57BL6J mice. Consistent with the superior tumourigenic and sphere formation potentials of the B16-GFP-D cells compared with the B16-GFP-M cells, the control mice (injected with dormant DMC-derived B16-GFP-D cells transfected with scrambled siRNA) developed faster-growing tumours in a significantly shorter time period than the mice injected with the corresponding maternal B16-GFP-M cells (Fig. 5a,b). Importantly, the transient siRNA-mediated down-regulation of GILZ in the B16-GFP-D cells, which was effective in the sphere cultures for a minimum of 7 days (Fig. 4c), significantly delayed the appearance of tumours (Fig. 5a) and reduced the tumour size (Fig. 5b). These results indicate that GILZ KD temporally induced the quiescence of dormant DMC-derived tumour-initiating MeSCs. Interestingly, qRT-PCR analysis of *Tsc22d3* expression in ultimately developed tumours revealed that they exhibited GILZ re-expression and that the tumours initiated by dormant DMC-derived MeSCs, particularly by GILZ-silenced MeSCs, expressed higher levels of GILZ mRNA than the corresponding maternal MeSCs (Fig. 5c). These data have demonstrated that GILZ controls the quiescence of slow-cycling MeSCs and that it is required for their reactivation and tumour or sphere initiation. These findings are supported by the data obtained using human HBL H2B-GFP melanoma cells, which revealed that GILZ KD significantly delayed the capacity of these cells to initiate tumours in SCID mice but increased their tumourigenic potential (Fig. 5d). Overall, these results demonstrate that *in vivo*, as *in vitro*, GILZ repression in human and dormant murine MeSCs induces cellular quiescence, contributing to melanoma dormancy and GILZ re-expression to promote MeSC reactivation and tumour development.

### Entry of dormant cells into G0 quiescence in response to GILZ down-regulation involves FOXO3A/p21CIP1 signalling

**Figure 6:**
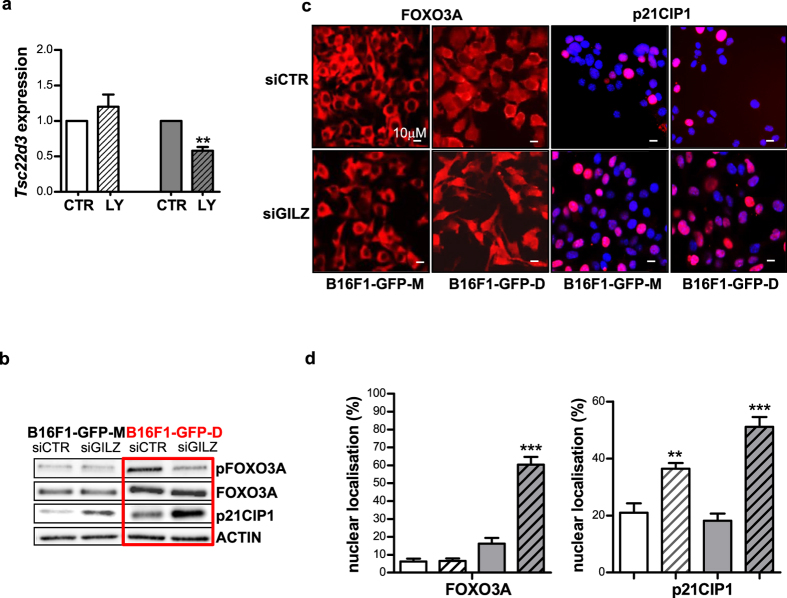
Down-regulation of GILZ induced regulators of quiescence. (**a**) Inactivation of the PI3K/AKT pathway via incubation in10 μM LY294002 (LY) for 24 h inhibited *Tsc22d3* expression in dormant DMC-derived B16-F1-GFP-D (right) and B16-F1-GFP-M cells (left) **p < 0.01 (n = 3). (**b**) Western blot analysis of relevant proteins affected by GILZ down-regulation in the maternal and dormant cell-derived cell lines; pFOXO3A denotes phosphorylated FOXO3A. (**c**) Down-regulation of GILZ affects the subcellular localisation of quiescence-related factors. Representative immunofluorescence images generated using anti-p21CIP1 and anti-FOXO3A (red) antibodies are shown. Hoechst 33342 (blue) was used for nuclear counterstaining. (**d**) Quantification by counting of the immunostained nuclei shown in (**c**); maternal cells (white), dormant cells (grey), GILZ KD (stripes); 4–15 fields were counted by 3 independent investigators. *p < 0.05; ***p < 0.001.

To obtain insights into the molecular mechanisms controlling cellular quiescence associated with GILZ inactivation, we first analysed an important downstream target of GILZ, the PI3K/AKT signalling pathway^18^,^19^,^21^, which has been implicated in the control of human MeSCs^4^,^12^,^13^,^15^ and tumour cell dormancy^42^. A key substrate of this pathway is FOXO3A, and its target is p21CIP1 cyclin-dependent kinase inhibitor, which control cellular quiescence^16^,^43^,^44^. As illustrated in Fig. 6a, the net result of inhibition of the PI3K/AKT pathway via LY294002 was the selective down-regulation of *Tsc22d3* expression in dormant DMC-derived B16F1-GFP-D cells. The siRNA-mediated repression of GILZ in these cells diminished FOXO3A phosphorylation (pFOXO3A), a sensitive indicator of AKT activity^45^, and increased the p21CIP1 protein level (Fig. 6b). This finding implicates GILZ as a mediator of FOXO3A inactivation and consequently a suppressor of p21CIP1 expression in dormant cells. In maternal B16F1-GFP-M cells, GILZ KD also increased p21CIP1 expression, but FOXO3A phosphorylation was unaffected. Therefore, it appears that in B16F1-GFP-M cells, GILZ regulates p21CIP1 in a FOXO3A-independent manner.

Active nuclear FOXO3A induces GILZ, which in turn negatively regulates its own expression by promoting the nuclear exclusion of FOXO3A^20^,^21^. Thus, GILZ KD should prevent the nuclear exclusion of FOXO3A in dormant DMC-derived cells but not in maternal cells. Indeed, the immunofluorescence data (Fig. 6c,d) indicated that GILZ KD selectively increased the pool of B16F1-GFP-D cells expressing nuclear FOXO3A. This result confirms that GILZ promotes FOXO3A nuclear exclusion in dormant DMC-derived melanoma cells, as in T-cells^20^,^21^, thereby validating that GILZ is a dormant cell-specific deactivator of FOXO3A in melanoma cells.

Nuclear FOXO3A functions as a transcription factor that controls diverse cellular processes, including cell fate determination, and it induces the cell cycle inhibitors p21CIP1, p27KIP1 and p57KIP2 for the maintenance of SC quiescence^43^,^46^,^47^. As expected, the increase in nuclear FOXO3A was directly proportional to the increase in nuclear p21CIP1 in the dormant GILZ-silenced DMC-derived cells but not in the maternal cells (Fig. 6c,d). This finding suggests that a molecular mechanism may exist by which dormant cells control their quiescent/activated states. Therefore, it appears that the capacity to readily repress PI3K/AKT-mediated GILZ expression disrupts the negative feedback on FOXO3A; this activity underlies a dormant cell-specific mechanism for controlling the quiescence/activation of murine MeSCs and the closely associated processes of tumour dormancy/relapse.

### GILZ down-regulation induces cellular quiescence in a dormancy model of human melanoma

**Figure 7:**
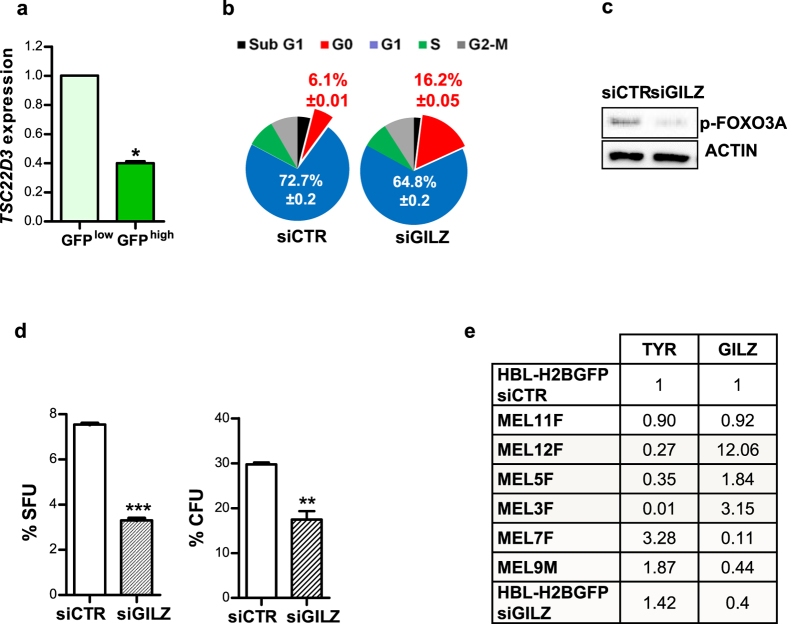
Down-regulation of GILZ induced cellular quiescence of human melanoma *in vitro* and *in vivo*. Human melanoma HBL-H2B-GFP cells were treated with tetracycline to induce the cell division-tracking H2B-GFP protein, a marker of quiescence, and were subsequently transfected transiently with control (siCTR) or GILZ-specific (siGILZ) siRNA. (**a**) qRT-PCR analysis of GILZ expression in FACS-sorted melanoma GFP^low^ fast-cycling cells and GFP^high^ quiescent cells. *p < 0.05 (n = 2). (**b**) Flow cytometry analysis of the cell cycle in control (siCTR) and GILZ down-regulated (siGILZ) human melanoma cells. (**c**) Immunoblots with an anti-phosphorylated (S253) FOXO3A (pFOXO3A) antibody after transfection of human melanoma HBL-H2B-GFP cells with control or human GILZ-specific siRNA. (**d**) Graphs showing the changes in the sphere-forming units (SFUs) and colony-forming units (CFUs) of human melanoma HBL cells after the down-regulation of GILZ expression. ***p < 0.001, **p < 0.01 (n = 3). (**e**) qRT-PCR analysis of relative *TYROSINASE* and *TSC22D3* expression in 6 human melanoma samples obtained from patients and in human melanoma HBL H2B-GFP cells treated with siCTR and siGILZ.

Because we found that GILZ-mediated signalling plays a significant role in controlling the quiescence/activation of dormant murine MeSCs, we questioned whether similar molecular events control quiescence and dormancy in human melanoma. In agreement with the relatively quiescent state of H2B-GFP label-retaining human MeSCs^31^,^32^, *TSC22D3* was significantly down-regulated in the FACS-sorted GFP^high^ subpopulation of human melanoma HBL-H2B-GFP cells (Fig. 7a). Cell cycle analysis of HBL H2B-GFP cells transiently transfected with human *TSC22D3*-specific siRNA demonstrated that, similar to mouse cells, the proportion of human melanoma cells entering the G0 phase of the cell cycle was increased with GILZ down-regulation (Fig. 7b and Supplementary Fig. S4). This increase was accompanied by a decreased level of inactive pFOXO3A (Fig. 7c) and subsequent reductions in the sphere-forming units (SFUs) and colony-forming units (CFUs) scored at one week after GILZ KD (Fig. 7d). The intriguing inverse correlation detected between the expression of *TSC22D3* and the melanocyte differentiation-specific *TYROSINASE (TYR)* gene (Fig. 7e) implies that GILZ, while stimulating the G0/G1-phase transition, may promote symmetric self-renewal in MeSCs by preventing their differentiation, thereby contributing to the enhanced tumourigenicity, as shown in Fig. 5. Thus, we have demonstrated that melanoma cells disseminate to multiple sites, even in the absence of a primary tumour, and that they persist in these sites as quiescent MeSCs that are resistant to immune responses. Their quiescence is associated with tumour dormancy *in vivo* and appears to be negatively regulated by the GILZ-mediated reversible inactivation of FOXO3A, a process that does not occur in maternal cells. Re-expression of GILZ and/or overexpression of genes belonging to the FOXO3A signalling circuit results in the reactivation of dormant cells, which may contribute to tumour relapse.

## Discussion

Primary tumours are rarely lethal themselves but often progress to lethal metastases. Clinical data and evidence from mouse models have revealed that metastatic cancer relapse occurs when dormant disseminated tumour cells survive primary treatment and become reactivated after varying periods of latency or tumour dormancy. The clinical observation of a high rate of donor melanoma transmission to immunosuppressed organ transplant patients^4^,^48^ is perhaps the best-documented example of tumour dormancy. Several studies have suggested that melanoma dormancy is associated with the survival of dormant solitary melanoma cells disseminated to distant organs^24^,^25^,^49^,^50^. We developed a clinically relevant syngeneic mouse model of melanoma dormancy to isolate these dormant DMCs and established first- and second-generation dormant cell-derived cell lines; to our knowledge, these are the only available cellular models of melanoma dormancy. Using these models, we identified several important molecular and cellular events related to melanoma dormancy. The following principles emerged from this work. 1) Dormant DMCs have the phenotypic and functional properties of MeSCs, which promote tumour dormancy in the quiescent state. 2) MeSCs disseminate early, maintain long-term quiescence and remain asymptomatic at 350 days after engraftment. 3) The immune system is not equally efficient in combating tumours versus disseminated cells. 4) In the brain environment, dormant DMC-derived cells become more prone to re-entering the cell cycle after another round of metastatic dormancy. 5) A molecular mechanism regulating cellular quiescence *in vitro* controls melanoma dormancy *in vivo*, and this finding provides evidence of a direct connection between the reversibility of CSC quiescence and the reversibility of tumour dormancy.

The ability to enter into reversible quiescence, which most likely developed as a default defence mechanism in response to intracellular and environmental stress signals, is the greatest survival advantage of SCs over their cycling progeny, aside from self-renewal^3^,^10^,^51^. CSC quiescence is responsible for the preservation of self-renewing abilities, resistance to anti-cancer therapy, evasion of immune responses and cancer relapse^8^,^10^,^14^,^25^,^27^,^33^. Thus, it is not surprising that, among the disseminated murine and human melanoma cells, the persistent cells were asymptomatic, quiescent MeSCs. These cells persisted without provoking adverse immune responses, even in vaccinated mice. The application of GM-CSF-mediated immunotherapy, which is known to stimulate CD8+ T-cells^22^,^23^,^52^, clearly prevented the development of primary tumours but was ineffective against quiescent MeSCs. The sole survival of MeSCs in tumour-free mice indicates that the GM-CSF-activated immune system eliminates the majority of strongly immunogenic melanoma tumour cells but not the less antigenic MeSCs^5^,^27^,^53^. The selective survival of less immunogenic CSCs is known to involve the CD8+ T-cell-mediated immuno-editing processes, which are responsible for the propagation of cancer cells without immune system interference^54^,^55^. Therefore, these processes could have contributed to the selective survival of quiescent MeSCs in our study. Their activation and propagation without triggering the immune system could explain the higher tumourigenic potential of the dormant DMC-derived cell line compared with the maternal cell lines.

A pressing and long-standing question is that of which mechanism controls MeSC quiescence and reactivation. Intriguingly, accumulated data indicate that the immune system may be responsible not only for tumour cell eradication and selection of tumour SCs but also for maintaining them in the dormant state^14^,^25^. For example, Eyles *et al*.^24^ have demonstrated that CD8+ T-cells inhibit metastatic progression and promote melanoma cell dormancy, which is elevated following CD8+ T-cell depletion. Additionally, Gerber *et al*.^56^ have shown that a higher Il-2 level in the tumour microenvironment enhances the number of IFN-γ-producing CD8+ T-cells, which attenuate B16 melanoma growth and stimulate cellular dormancy. Although our results do not directly demonstrate the dependence of MeSC quiescence on CD8+ T-cells, they are consistent with this possibility. Our GM-CSF-vaccinated mice, which were expected to have enhanced CD8+ T-cell activity^23^, were protected against tumour development but contained disseminated MeSCs that had been dormant for one year. These findings demonstrate that proliferating tumour cells and dormant MeSCs respond differently to immune signals, and they strongly support the dual activity of CD8+ T-cells, as observed by others^24^,^25^,^56^, which simultaneously maintain MeSC quiescence and eradicate more mature melanoma cells. Interestingly, we have shown that MeSC quiescence in dormant DMC-derived cell cultures is associated with a low level of GILZ, the mediator of glucocorticoid-mediaited immunosuppressive responses^20^,^21^. Thus, it appears that melanoma cell dormancy is maintained by at least two immune-related processes: intrinsically, by GILZ repression (our data), and extrinsically, by a process involving CD8+ T-cells^24^,^56^. MeSC reactivation and propagation require GILZ activity, as downregulation of GILZ inhibited these processes and the ultimately growing tumours and melanospheres expressed high levels of GILZ. The intriguing inverse relationship between the expression of *TSC22D3,* which encodes GILZ, and that of *TYR,* which encodes TYROSINASE, a melanoma differentiation marker as well as a strong tumour antigen, suggests that GILZ may prevent MeSC differentiation, thereby diminishing melanoma immunogenicity. This possibility is supported by the observation of larger MeSC pool (reflecting symmetric self-renewal) in the dormant cell-derived cultures and the increased tumourigenic potential of these MeSCs. In the future, it would be interesting to determine whether CD8+ T-cell-elicited signalling represses GILZ expression in dormant melanoma cells. This possibility appears very likely because pro-inflammatory cytokines, such as INF-γ and TNF, inhibit GILZ expression in T-cells^21^.

Our results provide the first evidence that GILZ suppression controls MeSC entry into quiescence *in vitro* and tumour dormancy *in vivo*. GILZ down-regulation induces re-entry of cycling MeSCs into the G0 phase of the cell cycle in dormant cell-derived cultures without significantly affecting maternal cells. We believe that the differences in the responses of these two cell lines to GILZ parallel the differences in the sizes of the MeSC pools; a smaller cell pool results in a smaller effect. It appears then that not the entire population but rather a subpopulation of native tumour cells has the capacity to enter a state of quiescence/dormancy and that dormancy-competent cells are present in each tumour cell line, albeit at different frequencies. Previously, we have shown that PI3K/AKT signalling is instrumental in controlling the size of the MeSC pool and the cycling activity of MeSCs by modulating FOXO3A functionality, among other factors^12^. The present findings have demonstrated that, in the context of the “dormant” phenotype, FOXO3A functionality is controlled by GILZ, which appears to mediate FOXO3A inactivation and consequently induce the down-regulation of the cyclin-dependent kinase inhibitor p21CIP1. It is conceivable that these events could promote the exit of MeSCs from quiescence in the G0 phase and drive their propagation, as both FOXO3A and p21CIP are known to maintain MeSC quiescence^8^,^16^,^43^. In addition, the results have shown that siRNA-mediated GILZ KD stimulates the G1-to-G0 phase transition, thereby repressing the formation of tumour-like spheres *in vitro* and delaying tumour development *in vivo*. These data establish direct links between GILZ and the G0 quiescent/activated status of MeSC as well as between GILZ and melanoma dormancy/relapse *in vivo*. These findings suggest that dormant disseminated tumour cells initiate metastatic tumour development. Although a direct link between the G0 quiescent/activated status of MeSCs and melanoma dormancy/relapse events remains to be experimentally confirmed, the correlation between dormant tumour cell reactivation and melanoma development in a case of transmission of donor melanoma via organ transplantation^4^,^48^ has spectacularly demonstrated the existence of this relationship.

Interestingly, the second generation of dormant cell-derived DMCs gained the ability to propagate and form colonies *in vitro.* This property of these cells was clearly associated with changes in the gene expression profile, including alterations in the expression of the *Ccnd2, Rxrb, Nupr1, Cdc25a* and *Id3* genes. This result may reflect the step-wise progression of melanoma to more aggressive metastatic disease after additional rounds of tumour dormancy, which may be promoted for example, by chemotherapy^9^,^10^,^33^,^57^, or by epigenetic changes induced by the microenvironment^2^,^57^. The progression of maternal cells to dormant DMC-derived cells with a more aggressi*v*e phenotype is related to the GILZ-mediated negative regulation of cellular quiescence and enlargement of the MeSC pool. A second step in this progression involves deregulation of the expression of cell cycle-controlling genes in brain DMC-derived cells. This interpretation strongly supports the evolving model of tumour dormancy^3^,^49^,^58^ and metastatic progression from “solitary” (long-term) cell dormancy in primary tumours to “micrometastatic” (short-term) and metastatic (rapid) dormancy as proposed by Giancotti^3^. Moreover, this conclusion is in agreement with the proposal by Crea *et al*.^57^ that the ability of CSCs to enter a dormant state is lost gradually with cancer progression, as characterised by the increasing inability of CSCs to differentiate, leading to the cellular homogeneity of their progeny. Collectively, our findings provide a foundation for future development of a strategy for cancer treatment that would differ according to the step of disease progression.

## Methods

### Cell culture

HBL human melanoma cells were a gift from Prof. Ghanem^59^, A375 human and B16F1 murine melanoma cells were acquired from ATCC (CRL-1619TM; CRL 6323TM). Cells were cultured as recommended by the suppliers. They were grown at 37 °C and 5% CO_2_ and were split 1:3 when 80% confluence was reached. All cells were re-fed with fresh media every other day. HBL-H2B-GFP cells were grown in the presence of selective antibiotics (400 μg/ml geneticin G418 and 0.5 μg/ml blasticidin). Reagents and the associated suppliers are listed in the Supplementary Information.

### Transfection and generation of transgenic B16F1 melanoma cells

The pcDNA3.1-Zeo plasmid was purchased from Invitrogen (France), and the mGM-CSF and GFP expression plasmids were obtained from Clontech (France). B16F1 melanoma cells were transfected using Lipofectamine. Geneticin (400 μg/ml) was used to select for the GFP-expressing vector, while zeocin (500 μg/ml) was used to select for the empty pcDNA3.1-Zeo and GM-CSF-expressing plasmids. GFP expression was analysed by flow cytometry and fluorescence microscopy. GM-CSF secretion was assessed using an ELISA murine GM-CSF kit (Diaclone, France). Large batches of selected B16F1-GFP, B16F1-pcDNA3.1-Zeo and B16F1-GMCSF clones were frozen until use. The selected B16F1-GM-CSF clone expressed 42 ng/ml of total GM-CSF per 10^6^ cells at 24 h after irradiation.

### Immunisation of mice and quantification of DMCs

Groups of between 10 and 30 C57BL6JRj immunocompetent female mice aged 8 to 10 weeks (Janvier Labs, France) were injected intradermally (i.d.) with 10^6^ irradiated (100 Gy from a ^137^Cr source) B16F1-GM-CSF vaccine cells or B16F1-pcDNA3.1-Zeo control cells. Cells were diluted in 100 μl PBS before injection. Each mouse received 2 injections per week for 19 days (Fig. 1a), and immunity was challenged at 7 days after the first injection by subcutaneous (s.c.) injection of 2 or 5 × 10^3^ B16F1-GFP challenge cells. Tumour-free mice in each group were sacrificed at 1 year after the challenge and were subsequently analysed for the presence of residual B16F1-GFP melanoma cells. All organs were removed, and RNA was extracted and transcribed into cDNA using random hexamers and a High Capacity Reverse Transcription Kit (Applied Biosystems, Life Technologies, France). qRT-PCR reactions were performed using specific primers and TaqMan fluorescent probes (Applied Biosystems, France) to detect GFP expression, and the relative expression ratios (using 18S ribosomal RNA as an endogenous control) were calculated using the comparative Ct (ΔΔ*C*_t_) method. To quantify the percentages of B16F1-GFP cells in the organs, ΔΔ*C*_t_ values were estimated based on the mixing of increasing quantities of B16F1-GFP cells (10^1^ to 10^6^) with a constant number of unlabelled cells.

### Isolation and characterisation of live persistent melanoma cells

To isolate persistent melanoma cells, immunised and control tumour-free mice were sacrificed at 1 year after challenge, the organs of interest (containing GFP+ cells, as detected by qRT-PCR) were removed and dissociated (4 mg/ml collagenase I and 3 mg/ml dispase), and GFP+ cells were sorted from cell mixtures from all of the organs from both the control and immunised mice using an EPICS-ALTRA sorter (Beckman Coulter). Skin samples included skin obtained from injection sites and surrounding areas. Cells were plated in DMEM containing 10% FBS and 1% antibiotics and were maintained in culture to obtain a new B16F1-GFP dormant DMC-derived (B16F1-GFP-D) cell line. Because the cell sorting was not exclusive and the GFP+ cells were still contaminated with GFP- host cells, we cloned the GFP+ cells after expansion. Thereafter, both clonal B16F1-GFP-D and maternal B16F1-GFP-M cells were propagated in DMEM containing 10% FBS and 1% antibiotics. To analyse the SC surface markers in the dormant DMC-derived melanoma cell line, single cell suspensions (2 × 10^5^ cells/100 μl) of B16F1-GFP-M and B16F1-GFP-D cells were incubated in growth medium with APC-conjugated anti-mouse CD133 (eBioscience, France) and CD24 (Santa Cruz Biotech) antibodies or a control isotype antibody for 30 min on ice. The antibodies were diluted 1:50. After incubation, the cells were rinsed with growth medium, centrifuged, resuspended in 500 μl of medium, and placed on ice before being analysed by flow cytometry. Dead and apoptotic cells were excluded according to cell morphology and PI (2 μg/ml, Sigma Aldrich, France) labelling preceding acquisition. Data were acquired with a CYAN flow cytometer (Beckman Coulter), and analysis was performed using Summit software. The APC and PI fluorescence intensities were recorded on the FL8 and FL3 channels, respectively.

### Tumourigenicity assay of the dormant melanoma cell line

To compare the tumourigenicity of the dormant DMC-derived melanoma cell line to that of the non-dormant maternal melanoma cell line, 200 and 5000 cells were injected (s.c.) into 8-week-old SCID CB17 (Pasteur Institute, Lille) or C57BL6JRj mice. When the mice developed melanoma, they were sacrificed, and the tumours and indicated organs were removed and dissociated to obtain cell suspensions for further analysis.

### siRNA-mediated gene KD

To evaluate the role of GILZ in dormancy, the dormant DMC-derived (B16F1-GFP-D) and maternal (B16F1-GFP-M) cell lines were transfected with 40 nM control or GILZ-specific siRNA (Applied Biosystems, France) to down-regulate GILZ expression. At 24 h post-transfection, cells were collected, and expression of *Tsc22d3* (human *TSC22D3*), which encodes GILZ, was assessed by qRT-PCR. The gene silencing efficiency was approximately 82% in all experiments, and the silencing lasted for a minimum of 7 days in sphere cultures. The cells were also assayed for their sphere formation capacities, cell cycle progression, protein expression and persistence in tissues or tumourigenic abilities *in vivo* and subjected to immunofluorescence analysis, as described above. Tumour growth was assessed at the indicated time points by performing bidimensional measurements with a calliper. Tumour volumes in mm^3^ were calculated using the following formula: width^2^xlengthx0.5. The same protocol was used for transfection of the human melanoma HBL-H2B-GFP cell line with the respective human siRNAs from Applied Biosystems.

### Cell cycle analysis

Cell suspensions were rinsed twice with PBS and fixed with cold 70% ethanol. After rinsing with cold PBS, cells were incubated with 50 μg/ml PI and 5 μg/ml RNase in PBS for 30 min at RT. To determine the percentage of cells in the G0 phase of the cell cycle, the cells were pre-incubated with an anti-mouse APC-conjugated Ki67 antibody (Abcam, France) or a matching isotype for 30 min on ice before the addition of PI and RNase and analysis by flow cytometry. The G0 cells were gated as Ki67-negative cells in the G0/G1 fraction of the cell cycle.

### RNA extraction, RT-PCR and qRT- PCR

RNA was extracted using an RNeasy Kit following the manufacturer’s protocol (Qiagen, Courtaboeuf, France) with optional DNase treatment and was stored at −80 °C. The following primers (Eurogentec, Belgium) were designed to amplify cDNA fragments ranging in size from 142 to 389 bp: *Ccnd2*, 5′-CGCAGTTTCCTATTTCAAG-3′; *Rxrb*, 5′-AGTACTGTCGCTATCAGAAG-3′; *Nupr1,* 5′-CTTTTGGAGAGAGCAGACTAG-3′; *Cdc25a*, 5′-CGATTCAGGTTTCTGTCTAG-3′; and *Id3*, 5′-CCTCCCTCTCATCTCTACTCTC-3′. For qRT-PCR, RNA was transcribed into cDNA using random hexamers and a High Capacity Reverse Transcription Kit (Applied Biosystems, USA). All qRT-PCR analyses were performed using TaqMan fluorescent probes (*TYROSINASE*, human *TSC22D3,* mouse *Tsc22d3*, GFP and 18S) provided by Applied Biosystems. The relative expression ratio for each gene was calculated using the ΔΔCt method. The calculated values represent the expression levels for each gene relative to the expression of 18S ribosomal RNA, which was used as an endogenous control.

### Melanosphere and colony formation assays, western blot and immunofluorescence analysis

Sphere formation, colony formation, western blot and immunofluorescence assays were performed as described previously^60^. For further details see the Supplementary Information.

### Human melanoma samples

Tumour samples (n = 6) were obtained from melanoma patients (Prof. Mortier L., Clinique de Dermatologie, CHRU de Lille, France) who had undergone tumour excision surgery.

### Statistical analyses

All results are expressed as the means ± SEM of at least 2 independent experiments with 3 replicates each. Comparisons between means were assessed using Student’s *t*-test for unpaired data. If unequal variance was observed, then Welch’s correction was applied. Statistical analyses were performed using GraphPad Prism software. A p-value of ≤0.05 was considered significant.

### Study approval

The storage and use of human biological samples were declared and performed according to the local Person’s Protection Committee and the ethical rules approved by the Department of Health of France (File No. DC-2008-642). Informed consent was obtained from all subjects. Animal experiments were performed in strict accordance with the recommendations of the Guide for the Care and Use of Laboratory Animals of the French National Institute for Medical Research (INSERM). The protocol was approved by the Committee on the Ethics of Animal Experiments of the Nord Pas-de-Calais (CEEA 09/2009). All efforts were made to minimise animal suffering.

## Additional Information

**How to cite this article**: Touil, Y. *et al*. Melanoma dormancy in a mouse model is linked to GILZ/FOXO3A-dependent quiescence of disseminated stem-like cells. *Sci. Rep.* **6**, 30405; doi: 10.1038/srep30405 (2016).

## Supplementary Material

Supplementary Information
