# Supplementary material for: Melanoma dormancy in a mouse model is linked to GILZ/FOXO3A-dependent quiescence of disseminated stem-like cells
Source: Sci Rep. 2016 Jul 28;6:30405. doi: 10.1038/srep30405 (PMC4964333; doi:10.1038/srep30405)
Supplement: Supplementary Information [file srep30405-s1.pdf]

Supplementary information

Melanoma dormancy in a mouse model is linked to GILZ/FOXO3A-dependent quiescence of disseminated stem-like cells

Authors: Yasmine Touil, Pascaline Segard, Pauline Ostyn, Severine Begard, Caroline Asspord, Raja El-Machhour, Bernadette Masselot, Jerome Vandomme, Pilar Flamenco, Thierry Idziorek, Martin Figeac, Pierre Formstecher, Bruno Quesnel and Renata Polakowska

Supplementary Methods:

Reagents: Propidium iodide (PI), trypsin/EDTA, and poly-2-hydroxyethylmetacrylate were purchased from Sigma-Aldrich (France), EGF was obtained from Stem Cells Biotech (Canada), and recombinant bFGF was acquired from PromoKine-PromoCell (Germany). Blasticidin, zeocin, geneticin (G418), collagenase type I, dispase and B27 supplement were obtained from Invitrogen (France). LY294002 was purchased from Calbiochem (France), DMEM and RPMI were obtained from Gibco®, Life Technologies™ (France), and FCS and 1% penicillin/streptomycin were purchased from Lonza (Belgium).

Melanosphere and colony-forming assays: Spheres were generated in 24-well plates. SFUs were estimated after 7 days. The spheres were counted under a microscope by 2 independent experimenters. For the self-renewal assay, primary spheres were recovered, dissociated by short (5 min) trypsinisation and re-plated at a clonal density of 1000 cells/ml. For the CFU assay, cells were plated at a clonal density of 100 cell/ml in 6-well plates.

Western blot analysis: Western blot analysis was performed using ready-to-use NuPAGE® 4%–12% Bis–Tris acrylamide gels according to the supplier's instructions (Invitrogen, France). The blots were probed with the appropriate primary antibodies against actin (Sigma-Aldrich, France), p21CIP1, GILZ (Santa Cruz Biotech), FOXO3A, and phosphorylated FOXO3A (pFOXO3A) at the S253 residue (Cell Signaling, USA). Then, they were incubated with a horseradish peroxidase-conjugated secondary antibody (Bio-Rad, France). Immunodetection was performed using an ECL+ chemiluminescence kit (Amersham).

Immunocytofluorescence: Cells were fixed with PAF and processed for immunohistochemistry according to standard procedures. The cells were incubated with primary rabbit anti-FOXO3A (dilution 1:100; Cell Signaling, USA) and anti-p21CIP1 antibodies (dilution 1:100, Santa Cruz Biotech) overnight at 4°C, followed by incubation with a secondary AlexaFluor 568 donkey anti-rabbit antibody (dilution 1:2,000; Invitrogen, France) for 1 h at RT. Hoechst 33342 (1 µg/ml) was used for nuclear counterstaining. All slides were mounted under coverslips and Vectashield Mounting Medium (Vector Laboratories) and imaged using a Leica fluorescence microscope.

Supplementary Figures:

Supplementary Figure S1: Immune systems of humanised mice prevented melanoma development but not dissemination of human melanoma label-retaining cells.

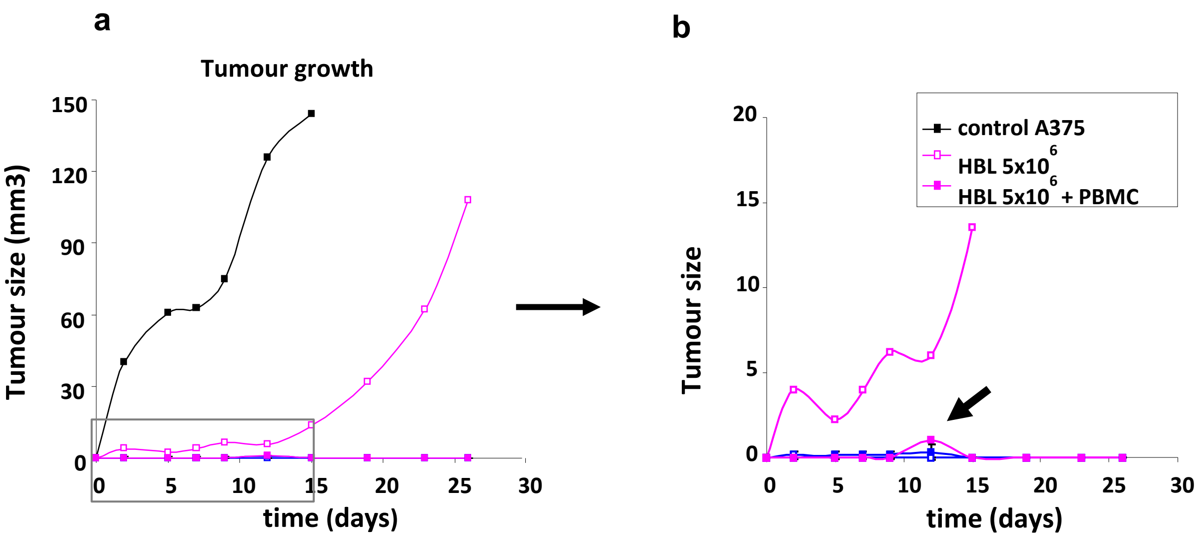

(**a** and **b**). To identify a direct link between dormancy and cellular quiescence, we used HBL human melanoma cells (HBL-H2B-GFP), which were stably transfected with an inducible tetracycline Histone 2B-GFP system (Ostyn P, et al. Cell Commun Signal. 2014;12:52) to discriminate quiescent or slow cycling GFP-positive cells from their fast cycling GFP-negative counterparts, and "humanised" NOD-SCID β2m-/- mice (Jackson Immuno Research Laboratories, Bar Harbor, USA) generated and bred at the Plateforme de Haute Technologie Animale (PHTA, France) as previously described (Asporд C, et al. PLoS ONE. 2010;5(5):e10458). Briefly, sub-lethally irradiated (120-150 Gy) "humanised" (receiving 50x10^{6} human PBMCs by i.p. injection) and "non-humanised" control mice were injected (s.c.) with 0.5-5x10^{6} GFP labelled HBL-H2B-GFP cells. Mice were injected (s.c.) with 10x10^{6} A375 human melanoma cells as a control for melanoma growth. The mice were sacrificed after one month, the organs were removed and enzymatically (4 mg/ml collagenase and 3 mg/ml dispase) dissociated, and the cell suspensions were analysed by qRT-PCR to detect the residual GFP-expressing quiescent cells. We subcutaneously injected the non-humanised control mice and the humanised mice with genetically modified HBL-H2B-GFP cells, which traces quiescent and slow cycling MeSCs. Whereas the control NOD-SCID ß2m-/- mice developed full-size tumours at the site of injection, the humanised mice developed only an initial small "bump" (arrow) that resolved 2 days later and remained disease-free for the next month. This result shows that the human immune cells could effectively eradicate the primary tumour cells at the site of injection within 2 days, although we could not exclude the more difficult-to-resist allogenic responses. The qRT-PCR analysis of skin, brain, liver, spleen, kidney and bone marrow cells showed that GFP was expressed in all organs from both the control and "humanised" mice except the skin of "humanised" mice (Supplementary Table S1). Importantly, the H2B-GFP expression identifies label-retaining, quiescent cancer stem cell. Thus, it appears that the human immune system is capable of inhibiting primary tumour growth but is unable to eliminate disseminated, GFP-retaining MeSCs. Framed area in **(a)** refers to the zoomed area in **(b)**.

Supplementary Figure S2: Comparative transcriptome analysis between dormant DMC-derived B16F1-GFP-D and maternal B16F1-GFP-M murine melanoma cell lines.

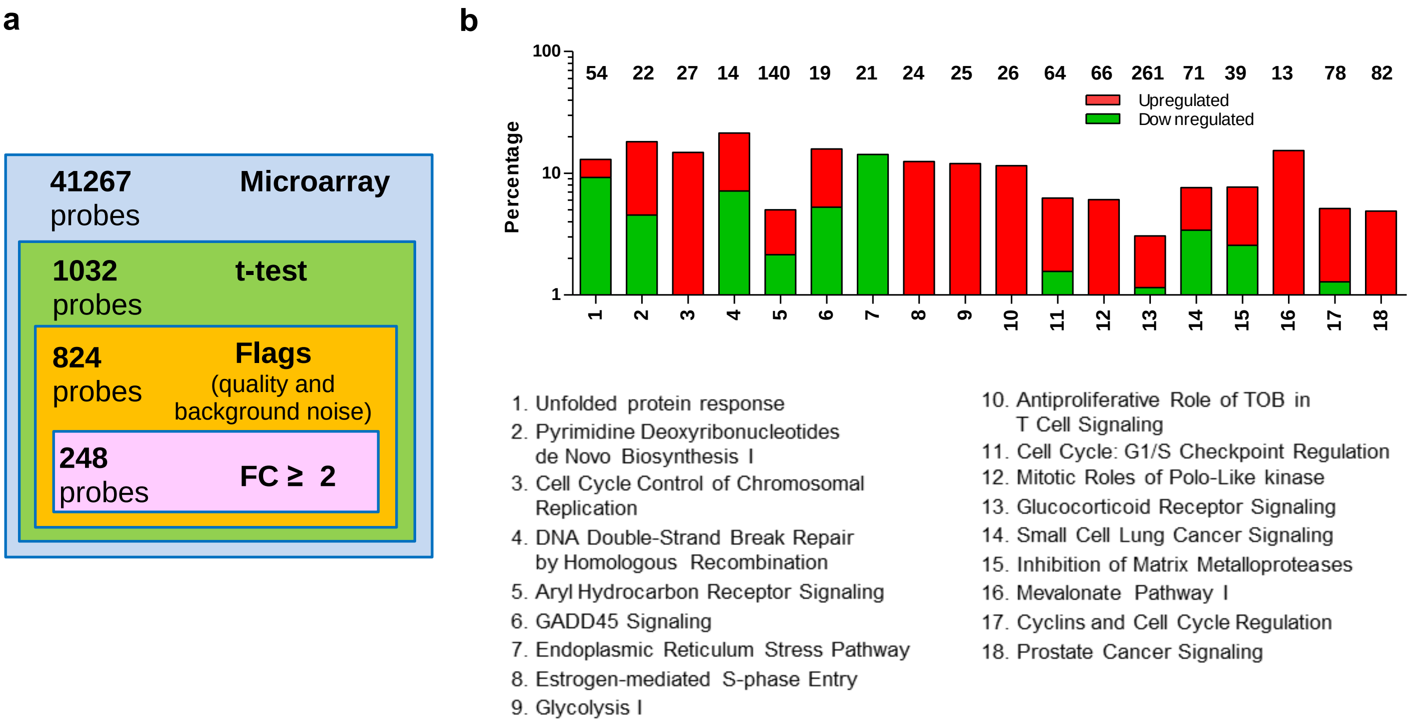

(a). Mouse Whole Genome Agilent 44K 60-mer oligonucleotide Microarrays were performed according to the Two-Colour Microarray-Based Gene Expression protocol (Agilent Technologies). Microarrays were scanned using the Agilent scanner G2505C and the Feature Extraction software (v10.5). The data were processed with GeneSpring (v10) for normalisation, filtering, and statistical analysis. The genes that were significantly up-regulated or down-regulated ($p<0.05$, t-test) were sorted using asymptotic P-value computations and Benjamini Hochberg FDR multiple testing corrections. The microarray data are available through GEO (accession number GSE69703). The expression of 248 genes was differentially regulated between B16F1-GFP-D and B16F1-GFP-M cell lines when the threshold was set at log≥2. **(b)**. The Ingenuity Pathway Network Analysis of deregulated genes in dormant DMC-derived B16F1-GFP-D versus mother B16F1-GFP-M cells. A set of 248 deregulated genes (229 mapped in Ingenuity Pathway Analysis) was obtained from the Agilent micro-array analysis of B16F1 GFP-D and B16F1 GFP-M cell transcripts. The networks are identified as sets of connected molecules that maximise their interconnectedness in the Ingenuity knowledge base. Only pathways with enrichment test p-value <= 0.01 (Fisher's exact right-tailed test) are displayed. Each gene in the network belonging to our set of deregulated genes was assembled according to disease and function. Bars illustrate the percent of molecules/genes deregulated in each pathway. The numbers on the top are the total number of molecules in each pathway. Up-regulated genes in B16F1 GFP-D cells are indicated by red and down-regulated genes are indicated by green.

Supplementary Figure S3: GILZ is downregulated in dormant DMC-derived murine melanoma cells.

a **b**

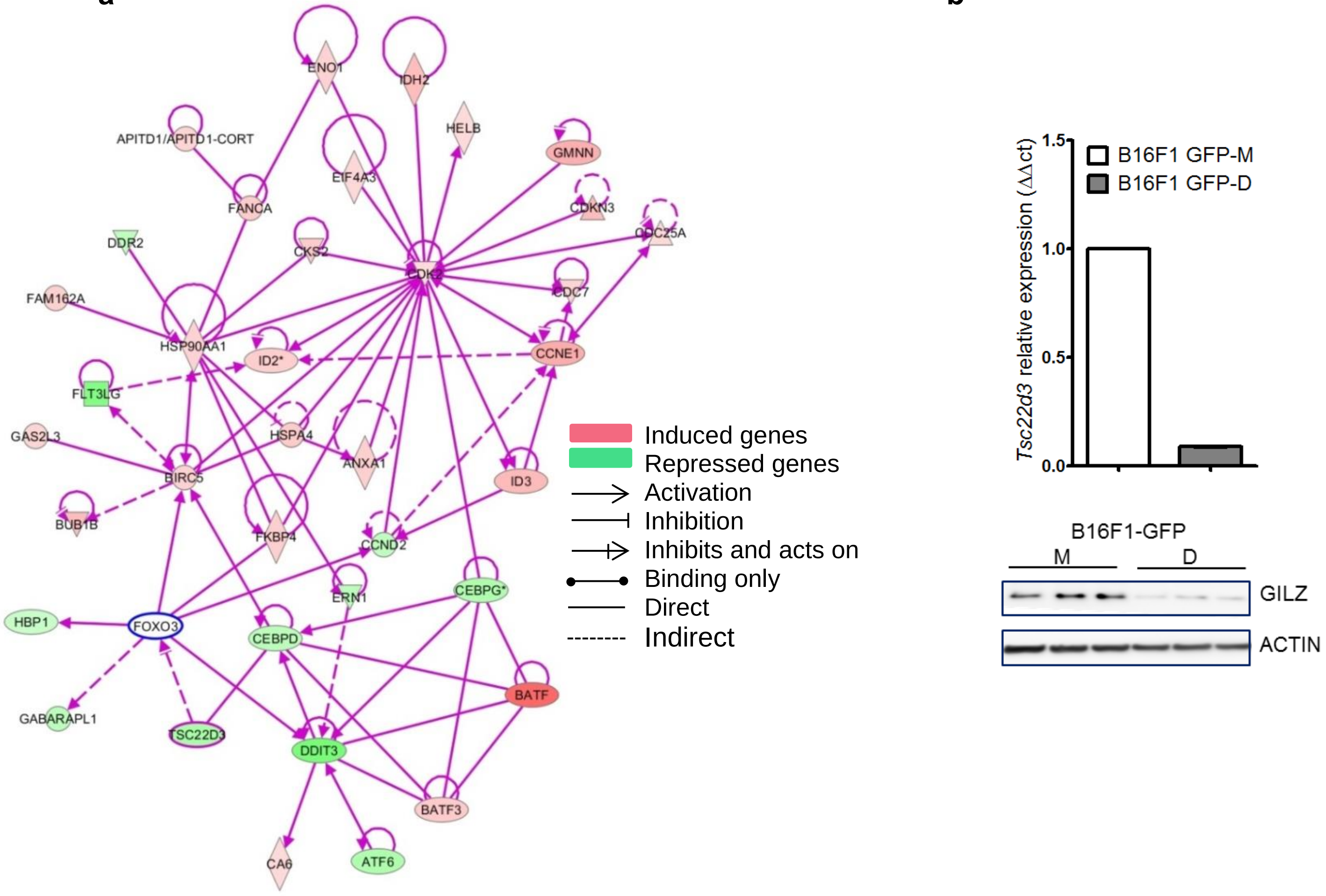

(a). The Ingenuity software-assisted identification of putative biochemical networks interconnected with the *Tsc22d3*. Green and red symbols indicate down- and up-regulated genes, respectively (dormant DMC-derived B16F1-GFP-D/maternal B16F1-GFP-M). Colour gradations indicate the fold-change levels. The scores associated with each Ingenuity Network are the –log base 10 values of the right-tailed Fisher exact test of the enrichment of our set of probes in the network. Only the Ingenuity networks with p-values of less than 0.01 are displayed. The black arrow points to the *Tsc22d3* gene encoding GILZ. **(b).** Among the down-regulated genes (log -3.29) in B16F1-GFP-D cells was *Tsc22d3*, which encodes the glucocorticoid-induced leucine zipper (GILZ) protein. qRT-PCR analyses of the B16F1-GFP-D and B16F1-GFP-M cell transcripts confirmed that the dormant DMC-derived cells expressed a considerably lower level of *Tsc22d3* transcripts (up) and GILZ protein (down) than maternal cells. Western blot shows levels of GILZ protein in 3 different samples of maternal (M) and dormant (D) cell cultures.

Supplementary Figure S4: siRNA-mediated down-regulation of GILZ increased the pool of human melanoma cells in the G0 phase of the cell cycle.

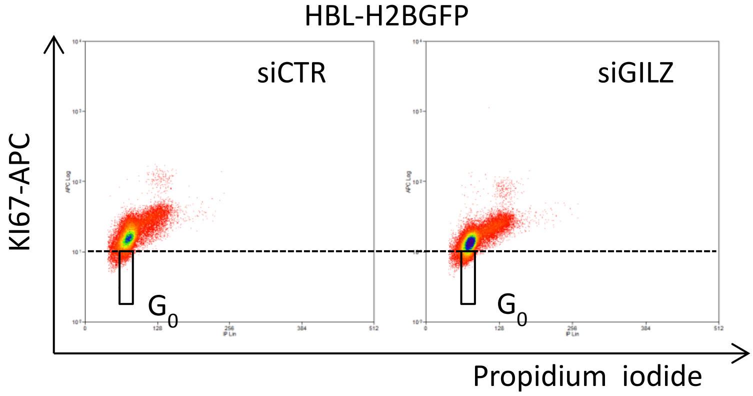

HBL-H2BGFP	SubG1	G0	G1	S	G2/M
siCTR	4.0±0.05	6.1±0.01	72.7±0.2	8.8±0.15	8.4±0.07
siGILZ	2.0±0.1	16.2±0.05	64.8±0.2	7.9±0.025	9.1±0.025

Flow cytometry cell cycle analysis of HBL-H2B-GFP human melanoma cells 24 h after transfection with control and GILZ-specific siRNA. The G0 cells were gated (box) as Ki67-negative cells in the G0/G1 phase of the cell cycle. For details, see the Methods. The table below shows the proportion of cells in each phase of the cell cycle. The sub-G1 fraction reflects the proportion of dead cells.

Supplementary Figure S5: GILZ knock-down (KD) did not significantly change the pool of dormant DMC-derived CD133+ cells but diminished their replication.

a

	EdU-	EdU+	CD133+	CD133+/EdU-	CD133+/EdU+
B16-M siCTR	62.69	37.31	1.69	0.40	1.29 (76.3%)
B16-M siGILZ	62.12	37.68	1.89	0.51	1.38 (73%)
B16-D siCTR	62.95	37.06	7.76	2.97	4.79 (61.7%)
B16-D siGILZ	72.48	27.52	8.44	4.39	4.05 (47.9%)

b

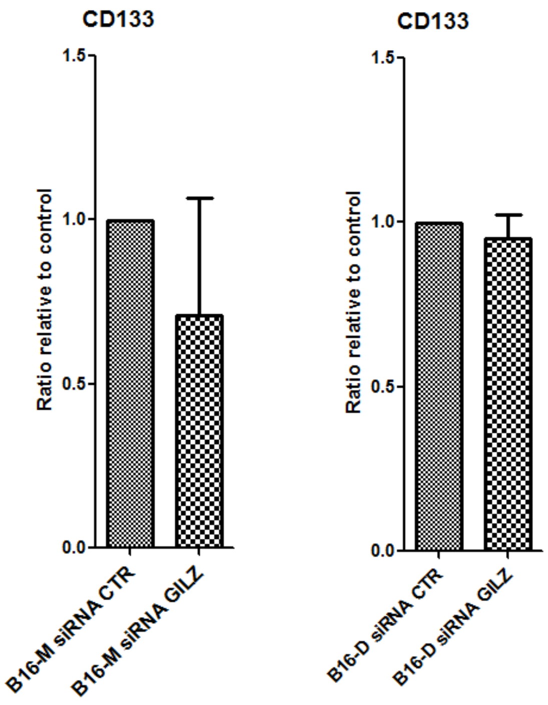

(a). GILZ KD expression inhibited proliferation of CD133+ « dormant » B16F1-GFP-D mouse melanoma stem-like cells. Flow cytometry analysis of maternal (B16-M) and dormant DMC-derived (B16-D) cells labelled with anti-CD133 Ab and EdU (ethynyldeoxyuridine) nucleoside using Click-iT® EdU proliferation assay. **(b).** Flow cytometry analysis of dormant DMC-derived B16F1-GFP-D (B16-D) and maternal B16F1-GFP-M (B16-M) cells 24h after transfection with control (siCTR) and *Tsc22d3*-specific (siGILZ) siRNAs and then labelled with anti-CD133 antibodies.

Supplementary Table S1. Vaccination with B16F1 melanoma cells expressing GM-CSF increased the number of tumour-free mice.

Vaccination	PcDNA 3.1	PcDNA3.1 + GM-CSF
Total # of mice	40	55
# of surviving mice	20	48
% of survival	50	87

The C57BL6/rj mice were vaccinated with irradiated B16F1 murine melanoma cells expressing recombinant mouse granulocyte-macrophage colony-stimulating factor (B16F1-GM-CSF). The mice pre-immunised at 7 days before the challenge and then immunised twice a week during the challenge with native B16F1-GFP cells exhibited significantly improved survival compared with the control mice, which were engrafted with irradiated B16F1-pcDNA3.1-Zeo cells for a minimum of 350 days. Mice were vaccinated as described in the Methods section. Please see the main text for details.

Supplementary Table S2: Dormant DMC-derived B16F1-GFP melanoma cells are present in tumour-free mice.

		Per cent of dormant melanoma cells per organ (Median)<br>(% of mice with dormant melanoma cells for the indicated organ)							
Mouse	#	Skin	Brain	Liver	Lung	Spleen	Kidney	Fat	BM
**Control** B16F1 pcDNA3.1	1	+	-	-	-	-	-	-	-
	2	+	+	-	-	-	-	-	-
	3	-	-	+	-	-	-	-	-
	%	**0.01**<br>22%;2/9	**0.001**<br>11%;1/9	**0.002**<br>11%;1/9	**0**<br>0/9	**0**<br>0/9	**0**<br>0/9	**0**<br>0/9	**0**<br>0/9
**Immunised** B16F1 GM-CSF	4	-	+	+	+	-	-	+	-
	5	-	+	+	-	-	-	-	-
	6	+	+	-	-	-	-	-	-
	7	+	+	-	-	-	-	-	-
	8	-	-	-	-	+	-	-	-
	9	-	-	-	-	-	+	-	-
	%	**0.25**<br>12%;2/17	**0.001**<br>23%;4/17	**0.0015**<br>12%;2/17	**0.01**<br>6%;1/17	**0.004**<br>6%;1/17	**0.01**<br>6%;1/17	**0.005**<br>6%;1/17	**0**<br>0/17

Organ distribution of B16F1-GFP-positive murine melanoma cells in tumour-free control and immunised mice. C57BL6/rj mice were injected i.d. twice a week for 19 days with either $1x10^6$ irradiated B16F1 cells expressing GM-CSF (immunised) or containing empty pcDNA3.1-Zeo vector (control). Mice were challenged with B16F1 cells expressing GFP (B16F1-GFP, s.c. injection) one week after the onset of the vaccination protocol and organs of tumour-free mice were analysed 350 days later by qRT-PCR. QRT-PCR reactions were performed using primers and TaqMan fluorescent probes to detect GFP expression and the relative expression ratios (using 18S ribosomal RNA as the endogenous control) were calculated (The median per cent of dormant cells per organ) using the relative quantitation ΔΔCt method, as described in the Methods section. BM=Bone Marrow

Supplementary Table S3: Quiescent human HBL H2B-GFP melanoma cells persisted in mouse organs.

SCID mice	Skin	Brain	Liver	Lung	Spleen	Kidney	BM
	(%x10^{-4} of H2B-GFP LRC)						
«Humanised »							
1	-	1.7	2	-	-	14	-
2	-	-	3.3	-	250	-	1.6
Control							
3	-	27	22	-	-	23	-
4	29	206	122	-	38	61	1.9

SCID (control) and "humanised" SCID mice were s.c injected with human HBL-H2B-GFP melanoma cells treated with tetracycline for 24h to induce H2B-GFP protein tracing label-retaining, relatively quiescent cells (LRC). One month later indicated organs were isolated and analysed for GFP expression by qRT-PCR. "-" =not detected BM=bone marrow.

Supplementary Table S4: Frequency of GFP+ maternal B16F1-GFP-M and dormant DMC-derived B16F1-GFP-D cells disseminated to different organs

Cells	Lungs	Brains	Livers
B16F1-GFP-M	0.38 ±0.1	12.0± 5.0	0.4±0.2
B16F1-GFP-DC	0.7 ± 0.1	9.0±1.8	1.7±0.6

B16F1-GFP-M and B16F1-GFP-D cells were transfected with the GILZ-specific and control siRNA. At 24 h post-transfection, 10,000 cells were engrafted into 5 syngeneic mice in each group. The tumour-bearing mice were sacrificed, and their lungs, livers and brains were analysed using qRT-PCR for the presence of GFP-positive tumour cells, as described in the Methods section. Note that the % of GFP-positive cells in the brain was higher than the % in the other organs. The data are expressed as the %x10^{-4} of GFP-positive cells per total number of cells isolated from each respective organ.
